# Comparing Venous vs. Capillary Blood Collection Methods for Proteomic Measurement in Peripheral Blood

**DOI:** 10.1002/prca.70007

**Published:** 2025-04-18

**Authors:** Mary Ni Lochlainn, Nathan J. Cheetham, Mario Falchi, Paolo Piazza, Claire J. Steves

**Affiliations:** ^1^ Department of Twin Research and Genetic Epidemiology King's College London London UK; ^2^ Nuffield Department of Medicine University of Oxford Oxford UK

**Keywords:** capillary, decentralised clinical trials, proteomics, remote studies, venous

## Abstract

**Background:**

Capillary blood collection has a number of advantages over venous collection, especially in the context of increasing decentralized clinical trials and field‐based testing. No studies are available comparing venous versus capillary blood collection for proteomics measurement. The aim of this study was to compare venous versus capillary blood collection methods for proteomic measurement in peripheral blood.

**Methods:**

The expression of 368 different proteins from the Olink Explore 384 Inflammation panel was measured in both venous and capillary blood samples collected from 22 individuals at a single time point. Protein levels from venous and capillary blood samples were compared with descriptive statistics and correlation calculations. Correlations were examined for a subset of proteins identified in recent reports as associated with morbidity and mortality.

**Results:**

Strong positive correlation (*r* ≥ 0.7) between protein concentrations measured in venous and capillary blood samples was observed for two in three proteins tested (215 of 327, 66%). A further 47 (14%) showed a moderate positive correlation (0.4 ≤ *r* < 0.7), with weak or very weak correlation (*r* < 0.4) observed for the remaining 65 (20%). Protein expression was consistently higher in capillary blood samples for proteins with lower correlation (*r* < 0.6) between sampling methods. Further work is required to understand the underlying reasons why proteins were consistently under‐expressed in venous samples/over‐expressed in capillary samples in a minority of proteins tested.

**Conclusions:**

Proteomic measurement utilising capillary blood collection provides very similar results to using venous blood collection. This is a promising sign for the validity and reliability of studies using capillary blood collection, including decentralised and remote studies.

**Abbreviations**
BD: Beckton Dickinson (trade name)
IQR: interquartile range
NPX: normalised Protein eXpression
PCR: polymerase chain reaction
STT: serum separating tube

## 1 Background

Proteomics is the study of the interactions, composition, structures and function of proteins and their cellular activities [1]. Proteomic data is increasingly used by researchers, as part of a ‘multi‐omics’ approach, where data including genomics, transcriptomics, microbiomics, metabolomics and others, are analysed to gain further understanding of the interactions between these biological entities (e.g., [2, 3]). With rapid advances in statistical and computing techniques for handling large datasets, and greater access to proteomic testing panels, the use of proteomic analysis in research is likely to increase. Indeed, recent studies have shown promise for use of proteomics in predicting future morbidity and mortality [2, 4–7], finding proteomics data to be competitive with or outperforming models built from polygenic risk scores, metabolomics, demographics or clinical markers in predicting diseases such as type 2 diabetes and dementia.

There is a lack of understanding of the systematic physiological differences between sampling sites. Further, the Covid‐19 pandemic has accelerated recent drives towards remotely delivered studies [8], point‐of‐care tests which give speedy results to aid clinical decision‐making, and a greater range of home testing kits which aim to empower patients in the management of their own healthcare. In order to collect blood samples in remotely delivered studies, where access to a trained phlebotomist was not feasible, some researchers opted to use capillary blood collected by participants [9, 10, 11, 12]. While venous collection is the gold standard for testing blood, capillary blood collection using a lancet is less invasive, cheaper, empowering and timesaving for participants, avoiding the need to travel to see a trained phlebotomy professional. It may also be preferable for patients with severe burns, those who are obese, or have a fear of needles and/or hospitals [13, 14]. Further, it removes the risk to the phlebotomist of exposure to a blood‐borne infection. Lastly, it involves a smaller quantity of blood and therefore a lower risk of anaemia, which is particularly pertinent in paediatric care. While there is limited data available on patient preferences, one study of 36 patients reported 94% preferred capillary to venous blood draw [15].

In spite of these advantages, there have been limited studies comparing capillary and venous blood collection techniques, notwithstanding calls from researchers for more work in this area [16]. Studies of coagulation assays have shown most routine clotting tests correlate well between the two sampling methods [17]. Examples of point of care capillary assays currently in clinical use include D‐dimer [18], international normalised ratio [19], lead levels [20] and haemoglobin [21]. No studies are available comparing venous versus capillary blood collection for proteomics measurement. The aim of this study was to compare venous versus capillary blood collection methods for proteomic measurement in peripheral blood.

## 2 Methods

### 2.1 Study Aim

This study aimed to compare proteomic signatures in peripheral blood via two collection methods: capillary blood and venous blood.

### 2.2 Study Design

In order to compare venous to capillary blood collection for proteomic analysis, individuals were recruited from the TwinsUK longitudinal cohort to have a capillary sample taken from a fingertip, and immediately afterwards a serum sample taken from a peripheral vein. Using these samples, proteomic measurements were carried out and the two sample collection types compared.

### 2.3 Study Setting and Participants

All participants were volunteers in the longitudinal TwinsUK study [22]. Eleven twin pairs (22 individuals) took part. Single twins were not included, as recruitment of twin pairs tends to improve participation rates in sub‐studies, and also allows for future analysis of the heritability of protein levels outside of the scope of this study. An opportunistic recruitment strategy was implemented whereby any twin pairs attending the department for a longitudinal twin visit were invited to take part from 26th September 2022 until the target number of twins was met on 4th October 2022.

### 2.4 Sample Collection and Processing

Participants were asked to fast the morning of their visit. They had not eaten since the evening before. Capillary samples were collected first from participants’ fingertip using a 500 µL STT capillary tube. BD microtainers were used, sourced from Fisher Scientific [23]. Venous samples were collected directly afterwards using standard phlebotomy techniques from antecubital site. Hands were not warmed for either sample. Serum samples were obtained using BD vacutainer serum separator tube vacutainer (gold cap). Both capillary and venous samples were left at room temperature for 30 min to ensure clotting. Samples were then centrifuged at 14,000 rpm for 3 min. Following this, the samples were serum aliquoted and frozen at −80°C immediately.

Serum samples were then prepared by thawing and aliquoting on PCR‐plates according to agreed layouts with Olink. Samples re‐frozen and shipped to the Technology Platforms lab in Oxford for processing. The concentration of 368 different proteins in the peripheral blood (40 µL per sample) of 22 individuals was measured using the Olink Explore 384 Inflammation panel on the same experimental plate [24]. The inflammation panel was chosen because of the known strong associations between inflammatory proteins and biological ageing, which is a key area of research for TwinsUK [25].

Olink library preparation and sequencing were performed using the Explore proximity extension assay (PEA) technology. PEA was performed as per the proteomic method previously described by Wik et al. [26]. Each assay has been extensively validated for the limit of detection, measurement ranges, precision, reproducibility and specificity, as detailed at https://olink.com/technology/what‐is‐pea. PEA uses high‐multiplex matched pairs of antibodies, each conjugated to a unique DNA oligonucleotide. Antibodies are incubated with their respective target proteins, facilitating specific binding. Upon hybridization of antibodies, DNA polymerase‐mediated extension generates a unique DNA barcode for each antibody‐target interaction wherever both specific antibodies recognise the same protein. The resulting DNA barcodes are then amplified using polymerase chain reaction (PCR), and Olink libraries purified using Agencourt AMPure XP magnetic beads to remove unwanted PCR byproducts. The prepared Olink libraries were then sequenced using a SP flow cell on the Illumina's NovaSeq 6000 platform. Finally, data underwent quality control checks, both in terms of assessing the reliability of each sequencing run and individual samples.

Relative protein concentration was given in terms of the Olink proprietary arbitrary unit, Normalised Protein eXpression (NPX). NPX is normalised based on inter and intra‐plate control samples and log2‐transformed [27].

### 2.5 Statistical Analysis

Results from control samples and assays (8 of 376) used for internal calibration were excluded. Results for 3 protein assays (BCL2L11, BID, MGLL) were excluded from analysis due to failing Olink quality control procedures. A further 38 proteins were excluded from analysis due to a significant number of participants (≥ 5 of 22) having protein concentrations below the baseline level of detection of the measurement, either for venous but not capillary blood samples (*n* = 11), capillary but not venous (*n* = 1), or in both sample types (*n* = 26), which would reduce the reliability of analyses.

To assess the similarity in protein concentration between blood sampling methods, the Pearson correlation coefficient was calculated for each protein in the panel, testing correlation between protein concentration from venous and capillary blood samples for the 22 participants. Additionally, within‐person differences in protein concentration between venous and capillary samples were calculated, where within‐person difference = NPX (Capillary)—NPX (Venous). The mean and standard deviation of these within‐person differences were calculated for each protein.

Analyses were performed using python v3.8.8 and packages: numpy v1.20.1, pandas v1.2.4, matplotlib v3.6.0, seaborn v0.11.2.

## 3 Results

### 3.1 Sample Characteristics

Participants were majority female sex, dizygotic, same‐sex twin pairs, aged over 50 years old (Table 1).

**TABLE 1:** Participant characteristics.

Variable	Metric	Value
Total participants	*N*	22
Families	*N*	11
Zygosity: Dizygotic	*N*	14
Zygosity: Monozygotic	*N*	8
Sex: Female	*N*	17
Sex: Male	*N*	5
Age (years)	Median	72
	IQR	52–75
	Range	24–75
Ethnic group: White ethnic groups	*N*	22

### 3.2 Correlation in Protein Concentration between Blood Sampling Methods

**FIGURE 1:**
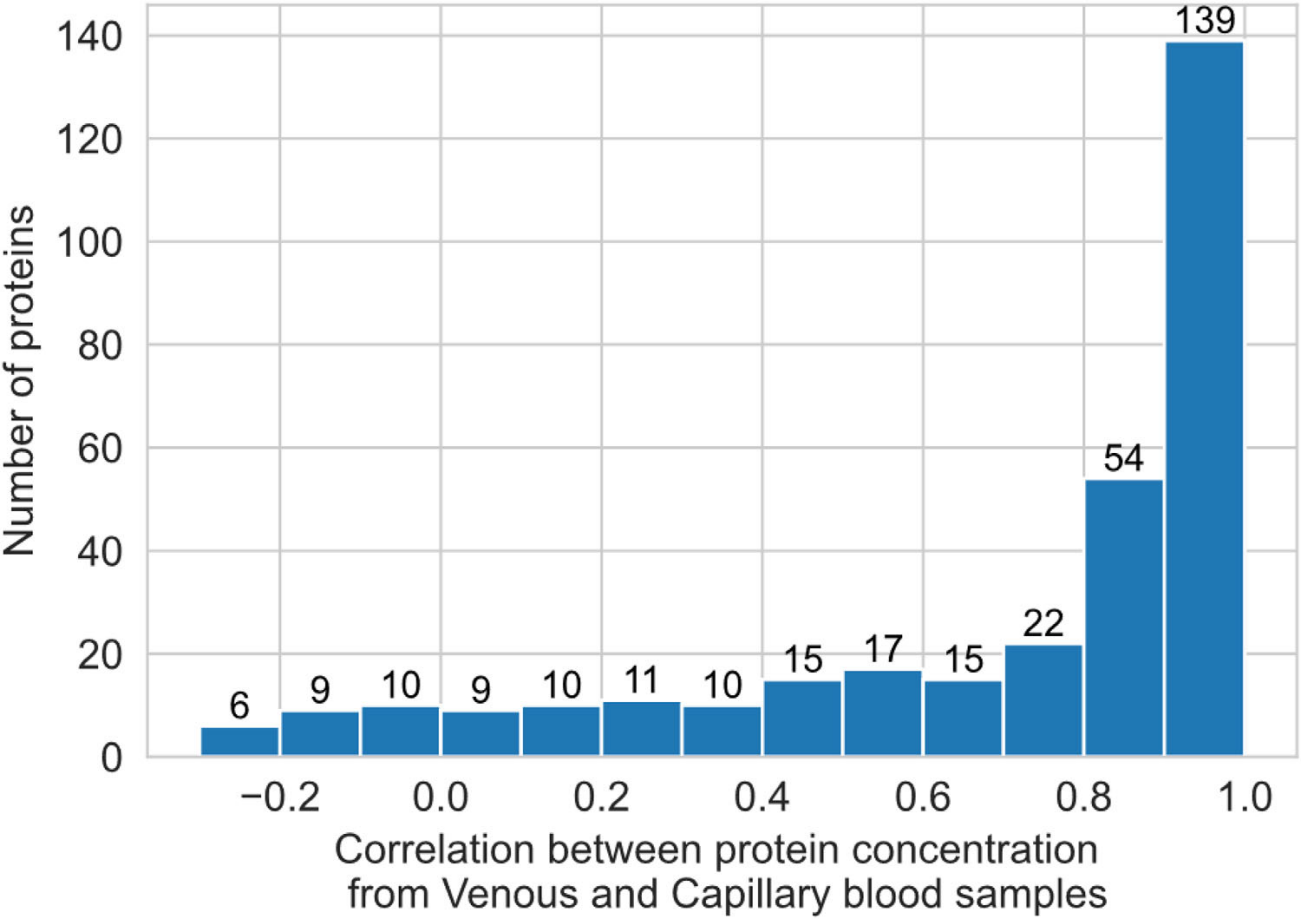
Histogram of correlation between protein concentration measured from venous and capillary blood samples. Data from 22 participants, testing 327 proteins from the Olink Explore 384 Inflammation panel. Correlation measured by the Pearson correlation coefficient. Relative protein concentration from Olink log2‐transformed arbitrary unit, NPX.

A strong positive correlation (*r* ≥ 0.7) between protein concentrations measured in venous and capillary blood samples was observed for two in three proteins tested (215 of 327, 66%) from the Olink Inflammation panel (Figure 1). A further 47 (14%) showed moderate positive correlation (0.4 ≤ *r* < 0.7), with weak or very weak correlation (*r* < 0.4) observed for the remaining 65 (20%). Full tabulation of correlation for each protein tested is given in Table S1.

**FIGURE 2:**
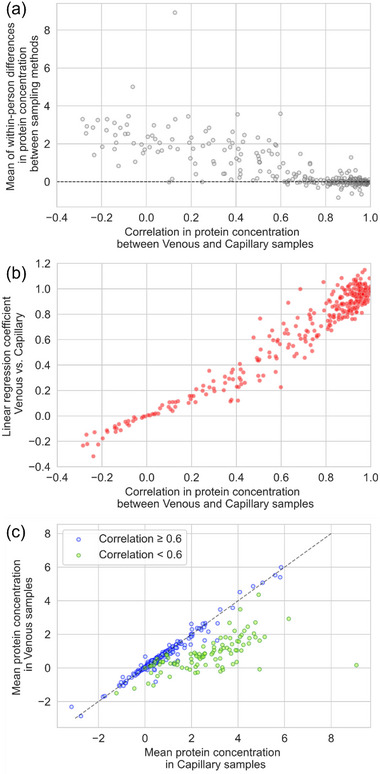
(a) Mean of the within‐person difference in protein concentration between venous and capillary blood sample, plotted against correlation between venous and capillary sample protein concentration. Correlation measured by the Pearson correlation coefficient. Relative protein concentration from Olink log2‐transformed arbitrary unit, NPX. (b) Linear regression coefficients of venous versus capillary protein concentrations, plotted against correlation between sampling methods. (c) Mean protein concentrations in venous versus capillary samples, grouped by coefficient of Pearson correlation between sampling methods.

Proteins with higher correlation between sampling methods also showed smaller mean of within‐person differences in protein concentration (Figure 2a) and linear regression coefficients closer to 1 in fits of venous versus capillary concentration (Figure 2b).

For samples with lower capillary‐venous correlation, the mean of within‐person difference was almost exclusively positive (Figure 2a), and linear regression coefficient <1 in fits of venous versus capillary concentration (Figure 2b), indicating that protein concentrations were higher in capillary blood samples than venous samples. Deviation in average protein concentration away from the line of equal concentrations in both sampling methods was observed for correlation coefficients under ≅ 0.6 (Figure 2c).

While lower levels of correlation were seen for proteins with lower levels of average signal relative to the limit of detection, proteins with low correlation between sampling methods were found across the range of protein expression levels, even at relatively high levels (Figure S1). When comparing the within‐person correlation between methods with reference data on abundance levels in humans (available for 318 of 327 proteins tested in this study), we saw a weak negative correlation (*r* = −0.25, *p* = 5 × 10^−6^), with higher abundance proteins tending to have slightly weaker within‐person correlations (Figure S2).

**FIGURE 3:**
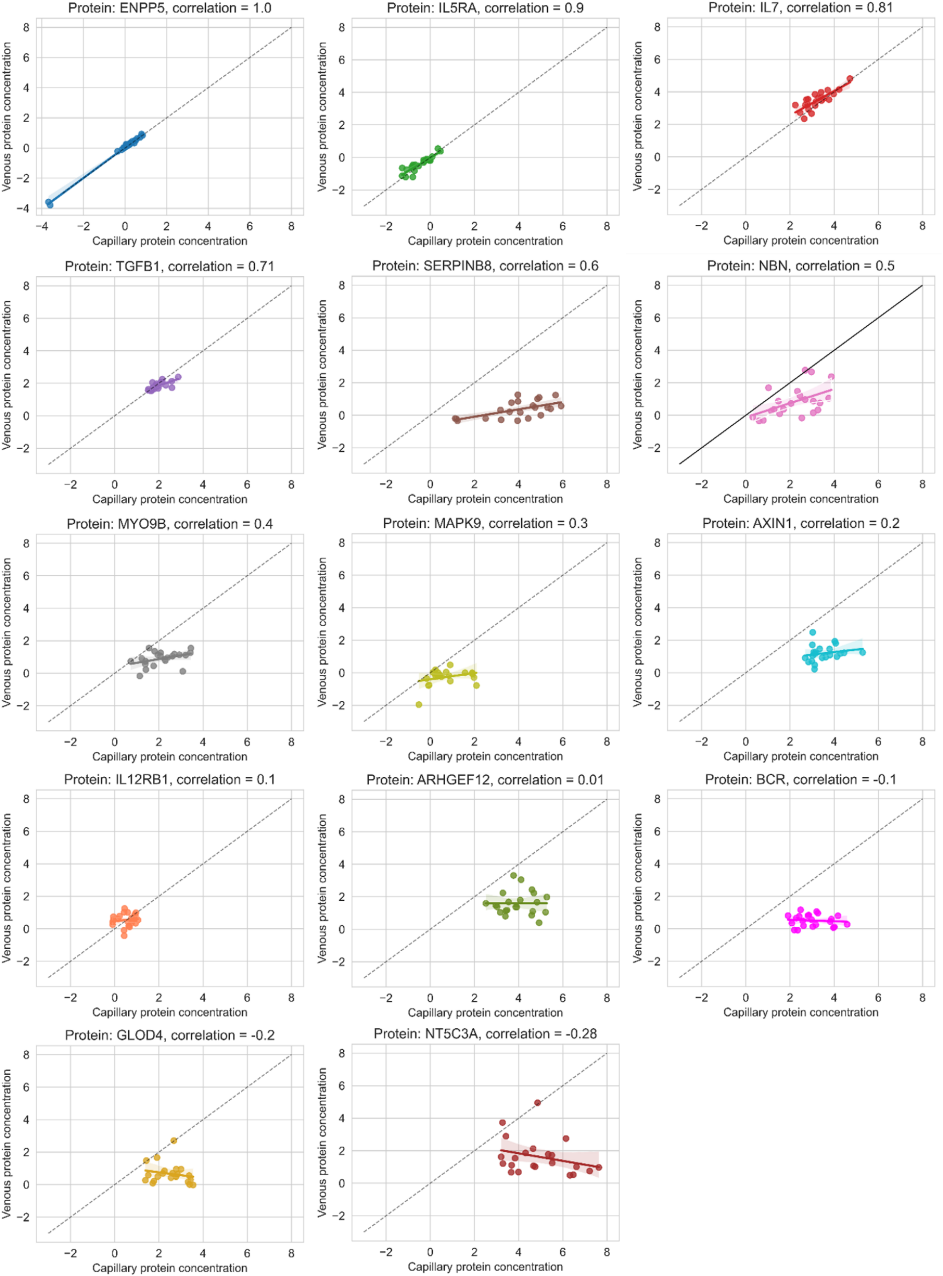
Example scatter plots of venous versus capillary protein concentration, ordered by Pearson correlation coefficient. Lines of equality (black dashed lines) and linear regression fits with 95% bootstrapped confidence intervals are shown for illustrative purposes.

The varying correlation between capillary and venous protein concentrations for individual proteins is illustrated in Figure 3.

### 3.3 Correlation among Proteins Associated with Morbidity and Mortality

As an applied example, we also highlight the venous‐capillary correlations for proteins previously identified as being associated with mortality and morbidity, exclusively from venous blood samples (Table 2) [5, 6, 7]. Proteins tested varied across studies, with 19 of 48 proteins identified as associated with 8 or more outcomes by Gadd et al. [6, 7], 21 of 169 proteins identified as predictive of one or more diseases by Carrasco‐Zanini et al. [5], and 3 of 34 proteins identified as in the top 1% most discriminatory by You et al. [4], also tested in our panel. For the 41 distinct proteins identified in the two previous reports that were available in our assay panel, the correlation between blood sampling methods was generally high, with 37 of 41 (90%) proteins having a Pearson correlation coefficient, *r* ≥ 0.7. Indeed, the correlation coefficient was higher for these proteins previously identified as associated with disease and mortality (median = 0.92, IQR = 0.87–0.96, range = 0.48–1.00), than across all 327 proteins tested in the panel (median = 0.86, IQR = 0.51–0.95, range = −0.28–1.00).

**TABLE 2:** Correlation between venous and capillary samples for proteins recently identified as associated with morbidity and mortality.

Assay	UniProt ID	Protein name	Correlation between venous and capillary samples	Morbidity & mortality predictors
ENPP5	Q9UJA9	Ectonucleotide pyrophosphatase/phosphodiesterase family member 5	0.998	Identified in You et al.
AGER	Q15109	Advanced glycosylation end product‐specific receptor	0.982	Identified in Carrasco et al.
NFASC	O94856	Neurofascin	0.980	Identified in Carrasco et al.
LAMP3	Q9UQV4	Lysosome‐associated membrane glycoprotein 3	0.979	Identified in Gadd et al.
LAIR1	Q6GTX8	Leukocyte‐associated immunoglobulin‐like receptor 1	0.978	Identified in Gadd et al.
CXCL9	Q07325	C‐X‐C motif chemokine 9	0.976	Identified in Gadd et al.
MZB1	Q8WU39	Marginal zone B‐ and B1‐cell‐specific protein	0.965	Identified in Gadd et al.
TNFRSF13B	O14836	Tumor necrosis factor receptor superfamily member 13B	0.958	Identified in Carrasco et al.
FCRL2	Q96LA5	Fc receptor‐like protein 2	0.957	Identified in Carrasco et al.
CLEC4A	Q9UMR7	C‐type lectin domain family 4 member A	0.957	Identified in Carrasco et al.
CSF1	P09603	Macrophage colony‐stimulating factor 1	0.956	Identified in Gadd et al.
TNFRSF4	P43489	Tumor necrosis factor receptor superfamily member 4	0.956	Identified in Gadd et al.
LILRB4	Q8NHJ6	Leukocyte immunoglobulin‐like receptor subfamily B member 4	0.954	Identified in Gadd et al.
SIGLEC10	Q96LC7	Sialic acid‐binding Ig‐like lectin 10	0.950	Identified in Carrasco et al.
CCL7	P80098	C‐C motif chemokine 7	0.950	Identified in Gadd et al.
COL9A1	P20849	Collagen alpha‐1(IX) chain	0.945	Identified in Carrasco et al.
CCL3	P10147	C‐C motif chemokine 3	0.944	Identified in Gadd et al.
TLR3	O15455	Toll‐like receptor 3	0.942	Identified in Carrasco et al.
TNF	P01375	Tumor necrosis factor	0.938	Identified in Gadd et al.
CD276	Q5ZPR3	CD276 antigen	0.929	Identified in Carrasco et al.
SLAMF7	Q9NQ25	SLAM family member 7	0.922	Identified in Carrasco et al.
LRRN1	Q6UXK5	Leucine‐rich repeat neuronal protein 1	0.919	Identified in Carrasco et al.
PRSS8	Q16651	Prostasin	0.914	Identified in Gadd et al.
PGF	P49763	Placenta growth factor	0.912	Identified in Gadd et al.
TNFSF13	O75888	Tumor necrosis factor ligand superfamily member 13	0.908	Identified in Gadd et al.
HSD11B1	P28845	Corticosteroid 11‐beta‐dehydrogenase isozyme 1	0.896	Identified in Carrasco et al.
EDAR	Q9UNE0	Tumor necrosis factor receptor superfamily member EDAR	0.896	Identified in Carrasco et al.
IL6	P05231	Interleukin‐6	0.894	Identified in Gadd & You
HGF	P14210	Hepatocyte growth factor	0.885	Identified in Gadd et al.
CXCL17	Q6UXB2	C‐X‐C motif chemokine 17	0.881	Identified in Carrasco & You
ITGA11	Q9UKX5	Integrin alpha‐11	0.872	Identified in Gadd et al.
MMP10	P09238	Stromelysin‐2	0.870	Identified in Carrasco et al.
LGALS9	O00182	Galectin‐9	0.859	Identified in Gadd et al.
LIFR	P42702	Leukemia inhibitory factor receptor	0.818	Identified in Carrasco et al.
SCG3	Q8WXD2	Secretogranin‐3	0.802	Identified in Carrasco et al.
TNFRSF14	Q92956	Tumor necrosis factor receptor superfamily member 14	0.741	Identified in Gadd et al.
PLAUR	Q03405	Urokinase plasminogen activator surface receptor	0.710	Identified in Gadd et al.
CD200	P41217	OX‐2 membrane glycoprotein	0.631	Identified in Carrasco et al.
PRELP	P51888	Prolargin	0.576	Identified in Carrasco et al.
TRIM21	P19474	E3 ubiquitin‐protein ligase TRIM21	0.498	Identified in Carrasco et al.
SLAMF1	Q13291	Signaling lymphocytic activation molecule	0.477	Identified in Carrasco et al.

## 4 Discussion

### 4.1 Key Points

Our study is the first to our knowledge to compare proteomic measurements between venous and capillary blood sample collection techniques for a large panel of inflammatory proteins.

The majority (66%) of proteins tested had high correlation (*r* ≥ 0.7) between blood sample collection methods. This internal within‐person consistency shows capillary samples as mostly consistent with the more common/standard venous blood sampling method used currently. Of the remaining third of samples, 14% showed moderately positive correlation, and 20% (65 of 327 proteins) had a weak correlation (*r* < 0.4) between the two sampling methods. Notably, for samples with lower capillary–venous correlation, the mean of within‐person difference was almost exclusively positive, indicating that protein concentrations remained related but were proportionally higher in capillary blood samples than venous samples.

Utilising three recent papers for examples of key proteins of clinical relevance, we report that 90% of the 41 identified proteins had a high correlation between venous and capillary samples. The correlation coefficient was higher for these proteins previously identified as associated with disease and mortality (median = 0.92, IQR = 0.87–0.96, range = 0.48–1.00), than across all 327 proteins tested in the panel (median = 0.86, IQR = 0.51–0.95, range = −0.28–1.00) (Mann‐Whitney *U*‐test comparing distributions, two‐sided *p* value = 0.02). This supports the idea that remote, self‐collected capillary samples retain clinically relevant value for proteomics profiling.

### 4.2 Interpretation

Our results, and those of others comparing levels of proteins and other biomarkers within venous and capillary samples [4, 5, 6, 7], suggest that while quantitative measurements for many biomarkers are independent of collection method, others have significant differences.

For proteins with correlation coefficient above 0.6, bias (mean difference between sampling methods) was close to 0, suggesting that differences between sampling methods were primarily due to random variability. However, for proteins with weaker correlation, the primary source of variation between the two sampling methods was a proportional dependence between expression in venous versus capillary samples, the scale of which varied for each protein but always with higher expression in capillary blood, rather than random variability.

The apparent over‐expression of proteins in capillary samples/under‐expression in venous samples observed for a minority of proteins may be explained by biological differences between blood sample collection modes and requires further investigation. Importantly, there are some differences between blood collected via venepuncture and via capillary skin prick. Capillary blood more closely resembles arterial blood and contains blood from arterioles, capillaries and veins [28]. While one may expect concentrations of metabolites or proteins to be similar across any artery in the body at a given time, the concentration in veins may be different due to uptake/release from the tissue beds close to the sampling site [29]. Thus, while the samples may be expected to be very similar, they are not a direct comparison of like with like.

As well as potential biological differences, differences in collection technique may also affect protein expression levels, with a good flow necessary to avoid coagulation of capillary samples. It is also necessary to discard the first 1–3 drops of capillary blood due to higher variability in these drops, and contamination with skin cells or tissue fluid [30]. If collected with the correct technique, haemolysis levels should be similar across both samples [15], however, differences in haemolysis between sampling methods may affect protein expression.

Lower signal‐to‐noise for some proteins does not appear to explain low correlation for certain proteins, since low correlation was seen even among proteins with relatively high protein expression well above the detectability threshold (Figure S1).

### 4.3 Strengths and Limitations

Compared to arterial and venous blood, capillary blood can be easily collected from a variety of sources (e.g. finger, earlobe tip, arm or heel), and can be done so by patients or study participants themselves, without significant training. Higher expression in capillary samples for a minority of proteins may indicate greater potential for proteomics profiling. A strength of our study is the comparison of venous and capillary samples taken at the same time, with closely replicated sample processing. However, results may not be generalisable to those of samples taken at home by participants, where others have found significant differences between samples collected at home which require transit and unsupervised collection, versus those collected in a clinical setting with supervision and rapid processing [31]. Sampling of twin pairs may reduce variation in protein levels between individuals wherever genetic factors contribute to blood protein levels, however as we focused on within‐person variation between blood sampling methods, we do not believe that this sampling strategy had a significant impact on our results.

Future studies could randomise order of capillary and venous collection, replicate sampling across multiple sites, and consider the inclusion of other sampling methods with utility for remotely delivered studies, such as dry blood spots as a collection technique [32]. Our study utilised an Olink panel of inflammatory proteins, and findings may differ with other panels. It should be noted that of 225 proteins identified as top predictors of morbidity and mortality by Gadd et al. [6, 7], Carrasco‐Zanini et al. [5] and You et al. [4], only 5 were identified by all three studies, despite all originating from the same sample and same proteomic panel (UK Biobank Pharma Proteomics Project [33]) and examining largely overlapping outcomes. This highlights challenges around the replication and interpretation of large‐scale omics association studies as proteomic studies develop further.

## 5 Conclusions

Thus, for the use of capillary samples in future research, more work is required to establish which measures can be reliably measured using capillary samples, including an understanding of any biological drivers of differences in protein expression in order to interpret results between studies using different venous versus capillary sampling. Perhaps most crucially, we need to understand whether associations between protein signatures and health outcomes vary according to the collection method used to collect blood samples.

## Author Contributions

Claire J. Steves and Mary Ni Lochlainn conceived the idea, designed the study, and acquired funding. Nathan J. Cheetham carried out the statistical analysis. Mary Ni Lochlainn and Nathan J. Cheetham wrote the paper. All authors reviewed and edited the paper.

## Ethics Statement

This study was carried out under TwinsUK BioBank ethics, approved by North West—Liverpool Central Research Ethics Committee (REC reference 19/NW/0187), IRAS ID 258513. This approval supersedes earlier approvals granted to TwinsUK by the St Thomas’ Hospital Research Ethics Committee, later London—Westminster Research Ethics Committee (REC reference EC04/015), which have now been subsumed within the TwinsUK BioBank. Written informed consent was obtained from all participants. All research therefore carried out in accordance with the ethical standards laid down in the 1964 Declaration of Helsinki and its later amendments.

## Conflicts of Interest

C.J.S. has consulted for Zoe Limited for work on the Zoe Health Study. All other authors declare no conflicts of interest.

## Supporting information



Supporting information

Supporting information

## Data Availability

Data are available on request from TwinsUK, please see https://twinsuk.ac.uk/resources‐for‐researchers/access‐our‐data/ for details.
