# Supplementary material for: Comparing Venous vs. Capillary Blood Collection Methods for Proteomic Measurement in Peripheral Blood
Source: Proteomics Clin Appl. 2025 Apr 18;19(4):e70007. doi: 10.1002/prca.70007 (PMC12278044; doi:10.1002/prca.70007)
Supplement: Supplementary file 1 — Supporting information [file PRCA-19-e70007-s002.docx]

Comparing venous versus capillary blood collection methods for proteomic measurement in peripheral blood

# Author list

Mary Ni Lochlainn^1*^^ and Nathan J Cheetham^1,*^, Mario Falchi^1^, Paolo Piazza^2^, Claire J Steves^1^

^*^ Authors contributed equally, ^ Corresponding author

# Author Affiliations

1. Department of Twin Research and Genetic Epidemiology, King’s College London, United Kingdom
2. Nuffield Department of Medicine, University of Oxford, United Kingdom

Correspondence to: Mary Ni Lochlainn, [Mary.ni_lochlainn@kcl.ac.uk](mailto:Mary.ni_lochlainn@kcl.ac.uk)

# Supplementary information

Table S1 is provided in a separate spreadsheet file.


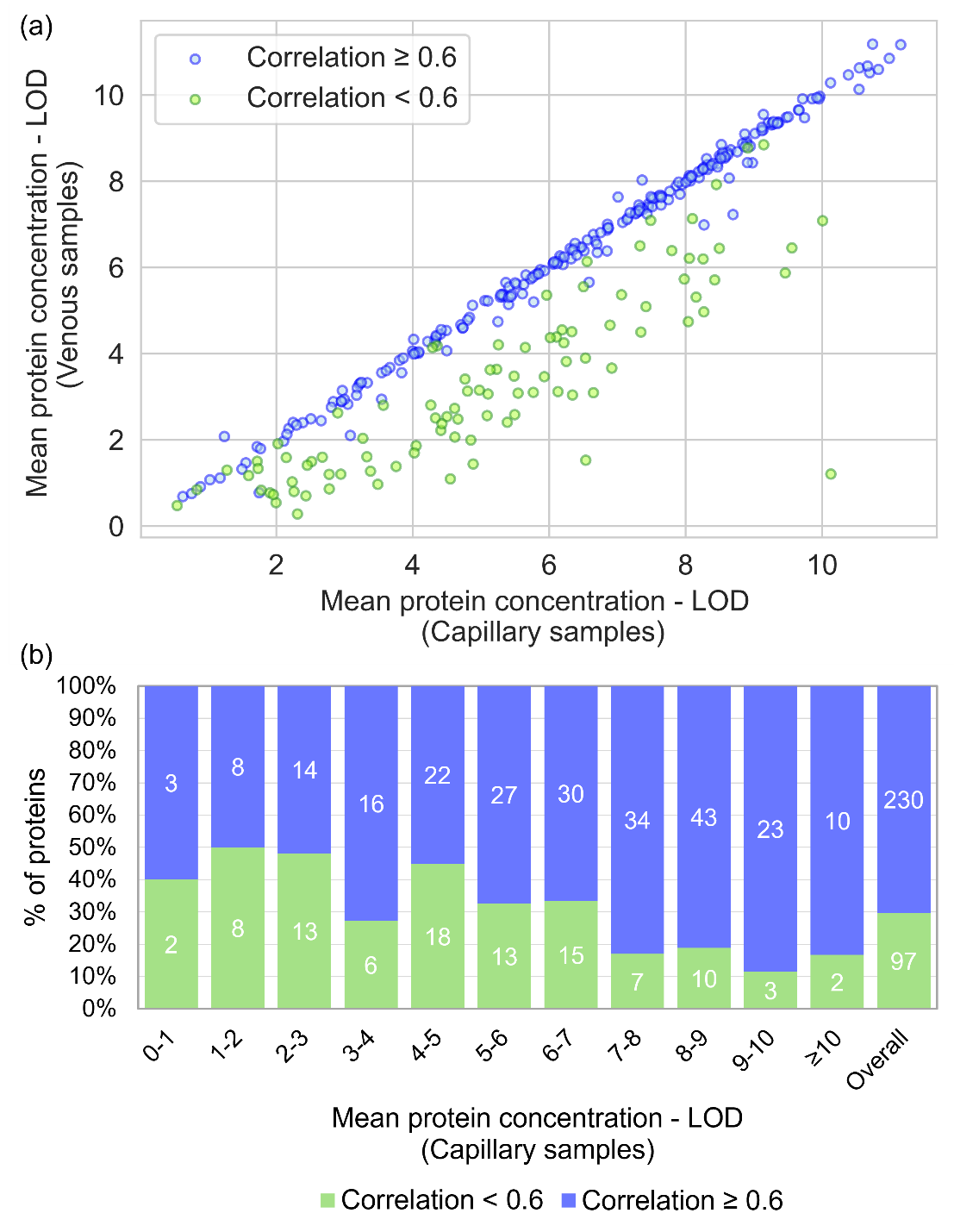


Figure S 1. (a) Mean protein concentration (Olink log2-transformed arbitrary unit, NPX) minus limit of detection (LOD) for given assay in Venous vs. Capillary samples, grouped by coefficient of Pearson correlation between sampling methods. (b) Frequency of proteins with within-person correlation between sampling methods of above or below r = 0.6, vs. mean protein concentration minus LOD in Capillary samples.


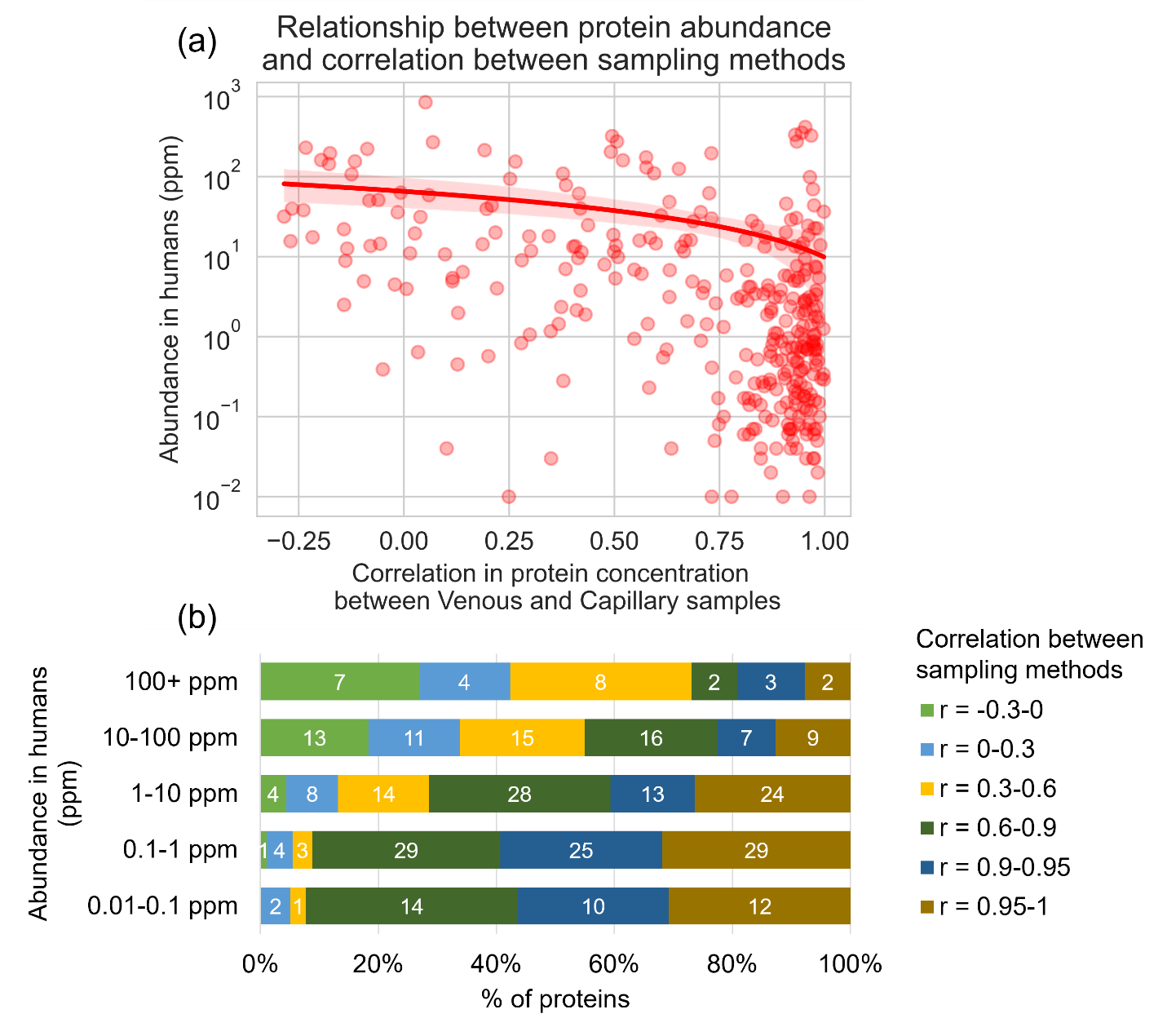


Figure S 2. (a) Protein abundance in humans (in parts per million, ppm) vs. coefficient of Pearson correlation between Venous and Capillary sampling methods. Linear regression fits with 95% bootstrapped confidence intervals are shown for illustrative purposes (b) Frequency of proteins after grouping by abundance in humans and Pearson coefficient of correlation between sampling methods.

The Pearson coefficient of correlation between abundance in humans and correlation between blood sampling methods was weakly negative, r = -0.25, p = 5 x10^-6^. Human abundances were obtained from the “H.sapiens - Whole organism (Integrated)” dataset of the PaxDb: Protein Abundance Database (<https://pax-db.org/>). Data from this dataset were available for 318 of 327 proteins analysed in this study.
